# Unifocal orofacial granulomatosis in retromolar mucosa: surgical treatment with Er,Cr:YSGG laser

**DOI:** 10.4317/jced.51301

**Published:** 2014-04-01

**Authors:** Pablo Castelo-Baz, Juan M. Seoane-Romero, Lucía García-Caballero, José M. Suárez-Peñaranda, María A. Romero-Méndez, Pablo Varela-Centelles

**Affiliations:** 1 DDS, MSc. Stomatology Department. School of Medicine and Dentistry. University of Santiago de Compostela. Spain; 2 DDS, MSc, PhD. Stomatology Department. School of Medicine and Dentistry. University of Santiago de Compostela. Spain; 3 MD, PhD. Pathology Department. School of Medicine and Dentistry. University of Santiago de Compostela. Spain; 4 MD, DDS, PhD. Stomatology Department. School of Medicine and Dentistry. University of Santiago de Compostela. Spain; 5 DDS, MMedSci, MPDH, PhD. Stomatology Department. School of Medicine and Dentistry. University of Santiago de Compostela. Spain

## Abstract

Orofacial granulomatosis is defined by permanent or recurrent swelling of orofacial tissues with different multiform and multifocal clinical patterns. An 11-year old boy presented with a 2-month history of mucosa enlargement. Intraoral examination revealed an erythematous, polylobulated, exophytic lesion with a smooth surface located in retromolar mucosa, non-tender and non-infiltratated to palpation. The diagnosis was inflammatory lesion compatible with pyogenic granuloma and laser excision was decided. Haematological parameters were within normal range, as well as chest Xrays. These findings lead to a diagnosis of non-symptomatic orofacial granulomatosis, whose early diagnosis can minimize the impact of systemic-related disorders, like Chron’s disease.

** Key words:**Laser, orofacial granulomatosis, childhood, oral lesions, diagnosis.

## Introduction

Orofacial granulomatosis (OFG) is a rare local disorder defined by permanent or recurrent swelling of orofacial tissues together with oral mucosal ulceration and a variety of orofacial characteristics (1). The chronic inflammation inherent to OFG often displays granulomas in the subepithelial stroma (2).

OFG etiology remains unclear although genetic background (atopia), allergy to foodstuff and/or dental materials (cinnamon, benzoate), infections (Mycobacteria spp, Saccharomyces cerevisiae, Borrelia burgdorferi) and immunological reactions (delayed-type hypersensitivity) have been involved in its causation (3).

Epidemiology of OFG is somehow unknown although no gender or race predilection has been described. This disorder seems to be more frequent in the second and third decades of life (4), with a reported prevalence around 0.8%, which is considered too high by some authors (5).

OFG may adopt different multiform and multifocal clinical patterns, including facial swelling (only or in combination with other signs), neurological manifestations, intraoral signs (6) (oral and aphtous ulcerations, linear ulceration, mucosal tags, cobblestone oral mucosa, and gingivitis and gingival enlargement) or perioral signs including lip swelling, vertical fissures on the lips, and angular cheilitis. Tongue swelling and tongue fissuring may also be caused by OFG (7). This lack of clinical specificity is accompanied by an unspecific histology, as the absence of non-caseificant granulomas does not exclude an OFG diagnosis when clinical features are suggestive of the disorder (6). These facts make atypical presentations –particularly those unifocal- a challenge for diagnosis and therapy. This paper reports on a very unusual presentation of OFG and its surgical treatment using an Er,Cr:YSGG laser.

## Case Report

**Figure 1:**
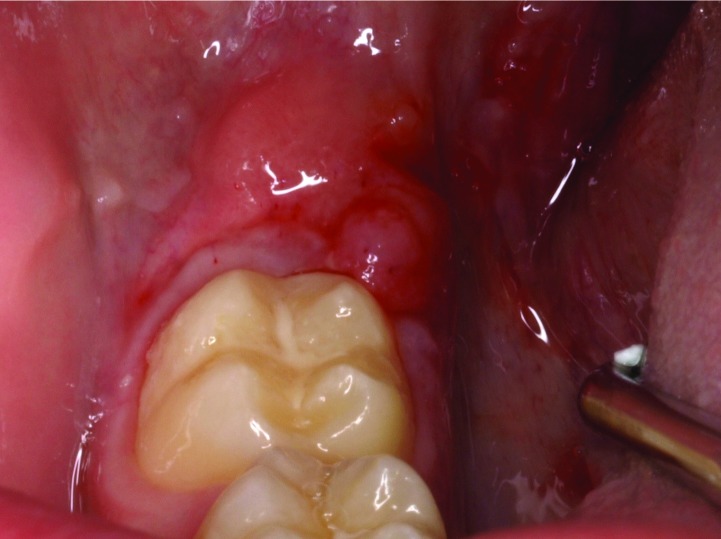
Clinical presentation of the lesion.

An 11-year old boy (C. B. L.) presents with a two month history of mucosa enlargement. Intraoral examination revealed an erythematous, polylobulated, exophytic lesion with a smooth surface located in retromolar mucosa (4th quadrant). The lesion was non-tender and non-infiltrated to palpation (Fig. 1). The patient was otherwise healthy and no neck nodes or other clinical or radiological signs could be identified. No history of allergy or drug intake was reported.

Based upon these findings, a diagnosis of inflammatory lesion compatible with pyogenic granuloma was made. Due to localized and exophytic nature of the lesion, surgical excision using an Er,Cr:YSGG laser (Waterlase MD. Biolase Technology Inc. San Clemente, CA, USA) working at a wavelength of 2780 nm. The device’s water-air cooling spray was set at a water/air proportion of 30/10% and the laser was operated at 2 W power, 20 (Hz), providing a density of energy per pulse of 35.7 J/cm2. A sapphire tip and a 600 mm diameter optic fiber were used.

After infiltrative and perilesional local anesthesia, the laser beam was perpendicularly directed to the lesion to outline the area to be excised (contact mode: slightly touching the mucosa). The specimen was then secured by a non-toothed Adson forceps and dissected once the deep margin was established. Haemostasia was ensured and the resulting wound was allowed to heal without suturing.

**Figure 2:**
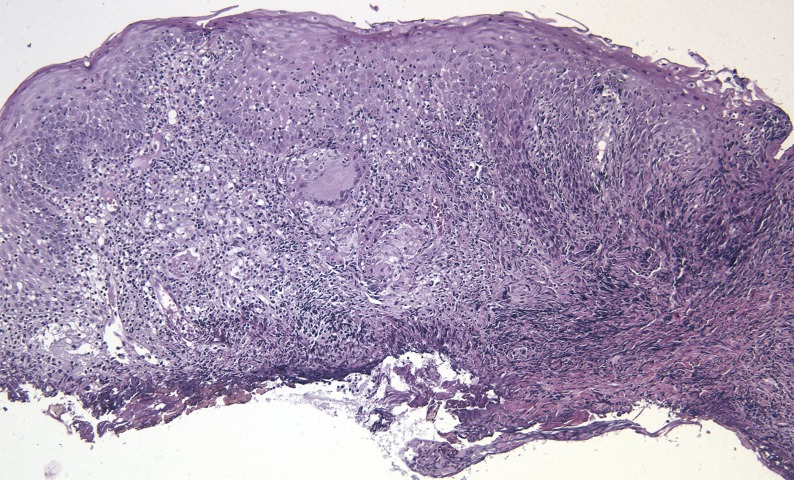
Histopathological examination revealed epithelial hyperplasia with a dense submucosal, mixed inflammatory infiltration that focally showed granulomatous features. Note the mild penetration in the tissue of the heat distortion induced by the laser.

**Figure 3:**
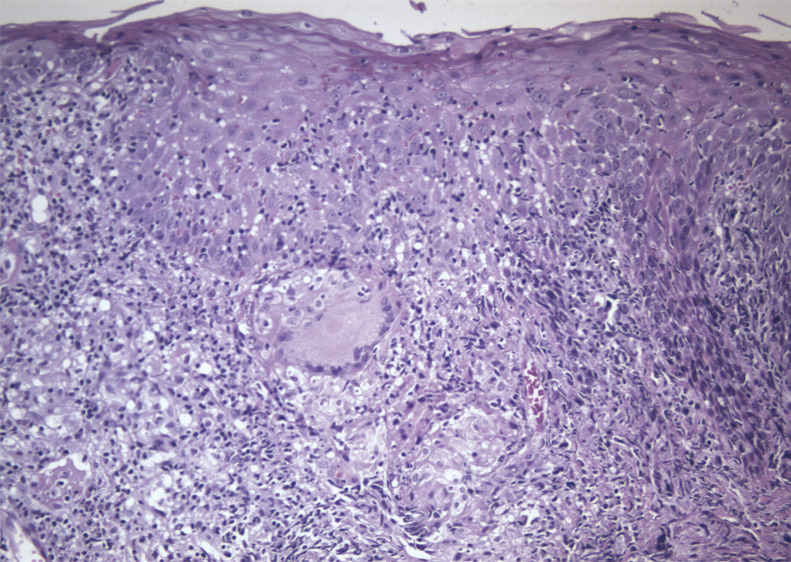
Granulomas were irregularly distributes in the specimen. They had giant multinucleated cells and did not show necrosis.

Pathological examination revealed moderate hyperplasia of the squamous epithelium, with basal regenerative changes without evidence of dysplasia. Superficial erosion was noted, accompanied by spongiosis and crust formation, but frank ulceration was not present. The submucosae showed a dense inflammatory process, with presence of lymphocytes, histiocytes, plasma cells and, to a lesser degree, neutrophils and eosinophils. Abundant epithelioid granulomas irregularly distributed along the specimen were also noted. They were sarcoid in type, with multinucleated giant cells, and did not show central necrosis. Interphase lesion along the basal layer of the epithelium was not noted and only isolated lymphocytes were noted in spongiotic areas (Figs. 2,3). PAS, Groccot and Ziehl-Nielsen stains failed to reveal the presence of microorganisms. Study with polarized light did not show birefingent foreign material either.

Hematological parameters (full blood count, hemoglobin, levels of C1INH, and angiotensin-converting enzyme level) studied a posteriori were all within normal range, as well as posteroanterior and lateral chest X-rays.

All these findings lead to a diagnosis of non-symptomatic OFG.

The surgical wound healed uneventfully within four weeks and no relapses or new lesions arose elsewhere after a six-month follow up period. The patient is currently being monitored for gastrointestinal symptoms.

This manuscript reports on a clinical case treated following established protocols, so ethical approval was not requested. The ethical principles for medical research involving human subjects (Helsinki Declaration) have been followed in this case report.

## Discussion

OFG diagnosis has to be established by excluding other disorders, like foreign-body reaction, sarcoidosis, tuberculosis, Chron’s disease (CD), hereditary angioedema, leprosy and deep mycosis (7). Atypical OFG may be under-diagnosed in up to 48% of cases (8) when initial biopsies do not disclose granulomas (3,6).

When granulomas are revealed in biopsy material, a differential diagnosis between other OFG and other granulomatous diseases is mandatory. Although pathological study cannot always reach a definitive diagnosis (e.g.: sarcoidosis’ and Chron’s disease’s granulomas share common features with OFG), it is able to exclude some disorders, as tuberculosis, on the basis certain frequent features (e.g.: caseating granulomas).

The OFG lesion in this 11 year-old patient arose following a very unusual pattern: non-ulcerated intraoral manifestation without facial swelling (6), depicting a unifocal variant of OFG.

The onset of OFG in pediatric age, as happens in this case, requires ruling out the possibility of a Chron’s disease because it is frequently linked to childhood and adolescent OFG (9). About 50% of children with OFG would develop Chron’s disease in the future (10), although intestinal lesions may take up to 10 years to develop (11). These cases of CD progressing from OFG, initially included lip swelling and are frequently related to atopia, allergic rhinitis or asthma (12), which were not found in this clinical case.

Diagnosis of Chron’s disease is based upon a combination of clinical, endoscopical, and radiological data as well as findings from surgical specimens (Lennard-Jones criteria). In this particular case, and bearing in mind the absence of hematological alterations and gastrointestinal symptoms, no gastrointestinal endoscopy was performed. This procedure should be restricted to those cases where clinical or laboratory features suggest the existence of CD (6).

OFG granulomatosis is a rare recurrent inflammatory disorder whose unclear etiopathogenesis hampers standardization of treatments based on sound scientific bases (3). Different therapeutical approaches have been proposed for multifocal OFG, including topical, intralesional or systemic corticosteroids, clofazimine, low-dose thalidomide, topical tacrolimus, TNF-α antagonists and low-level laser therapy. The response obtained to these therapies were highly variable (7,13).

Surgical management has been proposed for monosymptomatic presentations of the Merkelson-Rosenthal syndrome, particularly for the treatment of severe granulomatous cheilitis at a quiescent stage (3). The selection of a surgical approach using a Er,Cr:YSGG laser for this case was conditioned by the features of the lesion and the age of the patient. Despite laser irradiation with Er,Cr:YSGG laser (2W) has demonstrated promising results on oral mucosa in experimental models both in-vivo and ex-vivo (14,15), and to the best of our knowledge, this laser has never been used for the treatment of OFG. Er,Cr:YSGG lasers permit good vision of the surgical area with excellent surgical margins, seal lymphatics and nerve endings and minimize thermal artefacts for pathological analysis. These lasers also have additional advantages for pediatric patients, such as minimal hemorrhage and scarring and better postoperative period with less swelling and pain (14,15) when compared to other surgical alternatives.

Bearing in mind the clinical and pathological lack specificity of OFG, it is concluded that unifocal oral hyperplasic lesions should be considered as a potential clinical presentation of orofacial granulomatosis. When dealing with OFG in pediatric age, a long clinical follow-up period is mandatory to allow for an early diagnosis and minimization of the impact of systemic-related disorders. Er,Cr:YSGG laser could be considered a useful tool when a surgical approach is chosen for the management of this disorder.

## References

[1] Leão JC, Hodgson T, Scully C, Porter S (2004). Review article: orofacial granulomatosis. Aliment Pharmacol Ther.

[2] Hegarty A, Hodgson T, Porter S (2003). Thalidomide for the treatment of recalcitrant oral Crohn´s disease and orofacial granulomatosis. Oral Surg Oral Med Oral Pathol Oral Radiol Endod.

[3] Grave B, McCullough M, Wiesenfeld D (2009). Orofacial granulomatosis – a 20-year review. Oral Dis.

[4] Lourenço SV, Lobo AZ, Boggio P, Fezzi F, Sebastião A, Nico MM (2008). Gingival manifestations of orofacial granulomatosis. Arch Dermatol.

[5] Fitzpatrick L, Healy CM, McCartan BE, Flint SR, McCreary CE, Rogers S (2011). Patch testing for food-associated allergies in orofacial granulomatosis. J Oral Pathol Med.

[6] Al Johani KA, Moles DR, Hodgson TA, Porter SR, Fedele S (2010). Orofacial granulomatosis: Clinical features and long-term outcome of therapy. J Am Acad Dermatol.

[7] Mignogna MD, Fedele S, Lo Russo L, Lo Muzio L (2001). Orofacial granulomatosis whith gingival onset. J Clin Periodontol.

[8] Mignogna MD, Fedele S, Lo Russo L, Lo Muzio L (2003). The multiform and variable patterns of onset of orofacial granulomatosis. J Oral Pathol Med.

[9] Khouri JM, Bohane TD, Day AS (2005). Is orofacial granulomatosis in children a feature of Crohn’s disease?. Acta Paediatr.

[10] Rowland M, Fleming P, Bourke B (2010). Looking in the mouth for Crohn´s disease. Inflamm Bowel Dis.

[11] Saalman R, Mattsson U, Jontell M (2009). Orofacial granulomatosis in childhood-a clinical entity that may indicate Crohn´s disease as well as food allergy. Acta Paediatr.

[12] Kolho KL, Heiskanen K, Verkasalo M, Pitkäranta A (2011). Orofacial granulomatosis in children-a challenge for diagnosis and treatment. Int J Pediatr Otorhinolaryngol.

[13] Banks T, Gada S (2012). A comprehensive review of current treatments for granulomatous cheilitis. Br J Dermatol.

[14] Cercadillo-Ibarguren I, España-Tost A, Arnabat-Domínguez J, Valmaseda-Castellón E, Berini-Aytés L, Gay-Escoda C (2010). Histologic evaluation of thermal damage produced on soft tissues by CO2, Er,Cr:YSGG and diode lasers. Med Oral Patol Oral Cir Bucal.

[15] Gonzalez-Mosquera A, Seoane J, García-Caballero L, Lopez-Jornet P, García-Caballero T, Varela- Centelles P (2012). Er,CR: YSGG lasers induce fewer dysplastic-like epithelial artefacts than CO2 lasers: an in vivo experimental study on oral mucosa. Br J Oral Maxillofac Surg.

